# Comparative assessment of surface irregularities of enamel after bonding with different techniques followed by three composite removal methods: An atomic force microscopic study

**DOI:** 10.34172/joddd.2023.36867

**Published:** 2023-04-03

**Authors:** Safiya Sana, Mohammed Feroze Hussain, Rony T Kondody, Priyanka Jain

**Affiliations:** ^1^Department of Orthodontics & Dentofacial Orthopedics at Al-Badar Rural Dental College and Hospital, Gulbarga, India; ^2^Department of Orthodontics & Dentofacial Orthopaedics, Consultant in Bangalore, India; ^3^Department of Orthodontics and Dentofacial Orthopedics at Sri Rajiv Gandhi College of Dental Science, Bengaluru, India

**Keywords:** Debonding, Orthodontic brackets, Surface roughness

## Abstract

**Background.:**

To compare and assess the enamel surface roughness by Atomic Force Microscopy between ceramic and metal brackets after adhesive removal with 3 different methods.

**Methods.:**

90 extracted premolars were collected and divided equally into 3 groups G, Y, and R. With group G bonded with metallic brackets (using primer and Transbond XT), group Y with ceramic brackets (primer and Transbond XT), and group R with ceramic brackets (silane and Transbond XT). Each group was subdivided into 3 sub-groups (10 premolars each) based on the resin removal method as A: 12- flute tungsten carbide (TC) bur (high speed), B: 12- flute TC bur (low speed), and C: 30 flute TC bur (low speed). Surface roughness values were calculated and compared before bonding and also after adhesive removal by atomic force microscope (AFM). Measured data were analyzed using paired student t-test, ANOVA, and Tukey’s tests.

**Results.:**

Among the groups, group G showed increased surface roughness after debonding compared to group Y and group R, with Rq value showing a statistically significant difference (*P*<0.047). Whereas, within the subgroups, subgroup A (12-flute TC, high speed) with Rq showed increased surface roughness which was found to be statistically significant (*P*<0.042).

**Conclusion.:**

None of the adhesive removal methods was capable to restore the enamel to its earlier morphology; a statistically significant increase in surface roughness parameters was reported with a high-speed 12 flute TC bur for Rq and Rt.

## Introduction

Due to the increase in demand for orthodontic care, orthodontists preferred to provide their patients with better and aesthetically pleasing appliances and bonding techniques. The bonding technique was developed not only to reduce the metallic appearance of bands but also as an initiation to improvise the acceptance and aesthetic appearance of the orthodontic appliance.^1^

Although there are many advantages of the direct bonding technique in orthodontic therapy, like maintenance of healthy gingiva, better patient compliance and enhanced clinical effectiveness,^2^ the only disadvantage of this technique is that it can lead to irreversible alterations on the enamel surface.^3^

As more adults started taking orthodontic therapy, this promoted the development of ceramic brackets.^4^ Gross enamel damage was manifested after debonding with a few of the initial generations of ceramic brackets; the bonding strengths acquired with ceramic brackets are of chemical-mediated adhesion. A silane coupling agent containing a bi-functional molecule with a reactive silanol group at one end that binds firmly to glass and another molecule reacts with adhesive resins and undergoes polymerization, forming a cohesive bond with the adhesive material.^5,6^

With the advancement and improvement in newer generations of ceramic brackets, the possibility of enamel alterations is reduced. Although quantitative evidence to prove such a claim is very less.^4^ Ceramic bracket undergoes bond fracture primarily at the adhesive-enamel interface whereas in metallic brackets bond failures occur frequently at the adhesive-bracket interface. The bond strength of ceramic and adhesive is stronger than the bond strength of the adhesive and the enamel.^7^ During de-bonding, bracket failure, particularly with the ceramic bracket, occurs in adhesive failure ie.; at the resin and enamel but enamel fracture can also occur with metal bracket.^8^

With contemporary advancements in bonding materials (mechanical and physical properties), after debonding, the clean-up of residual resin has become a clinical challenge especially in maintaining the enamel integrity.^9^ Incomplete removal of these resin leftovers can lead to unesthetic discoloured surfaces on tooth and plaque accumulation due to irregular finished surfaces, ultimately leading to enamel demineralization.^10,11^

However, there is no universally approved protocol in the previous studies regarding this issue although routinely practised methods of residual adhesive removal from surface of enamel is by utilizing low-speed tungsten carbide (TC) bur accompanied by an appropriate polishing disc and subsequent polishing paste.^10,12-14^

Assessment of enamel surfaces following residual resin clean-up with different rotary instruments evaluated by scanning electron microscope (SEM) and other optical imaging scanners have proven to be unreliable and subjective and do not render the exact quantitative results.^15^ Atomic force microscope (AFM) assessment is one of the methods that utilize numerous scans at a high level of resolution and is proposed for surface analysis with irregularities at a nanoscale. Such quantitative assessment helps in a better comparative assessment of enamel destruction resulting from different debonding and composite clean-up methods.^16,17^ Furthermore, it has a lot of other advantages over other methods, for example, minimum specimen preparation, simultaneous 2Dimensional and 3Dimensional imaging and probability of re-examining the samples.^18,19^

 Hence this research aimed to compare and assessment of enamel surfaces subjected to various bonding (mechanical and chemical) and resin removal methods after debonding using an AFM.

## Methods

**Figure 1:**
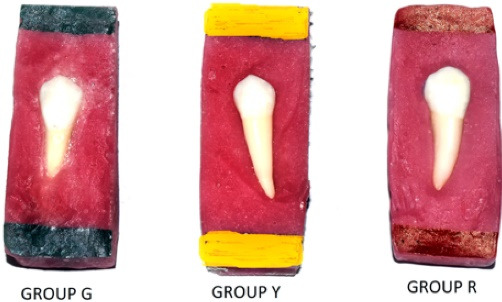
Extracted premolars mounted in a custom made acrylic blocks with the respective groups

Ninety extracted premolars (orthodontic purpose) were collected from patients in an age range of 15-20 years and preserved at room temperature in the water till they were prepared and analyzed. The selected teeth were intact with no caries or restorations, teeth with no hypo-calcifications or fluorosis and teeth with no visual cracks. The specimen teeth were embedded in custom-made blocks of acrylic resin and stored in 0.9% normal saline solution, later which were randomly categorized into three different groups based on colour codes as (G-green; Y-yellow; R-red) (Figure 1) to anticipate any association between the groups based on bonding method used.

###  Group division

- Group G = includes 30 premolars with metallic brackets (3M Unitek) bonded using primer and Transbond XT adhesive.
- Group Y = includes 30 premolars with ceramic brackets (3M Unitek) bonded using primer and Transbond XT adhesive.
- Group R = includes 30 premolars with ceramic brackets (3M Unitek) bonded using silane coupling agent and Transbond XT adhesive.

###  Assessment of enamel surface roughness

**Figure 2:**
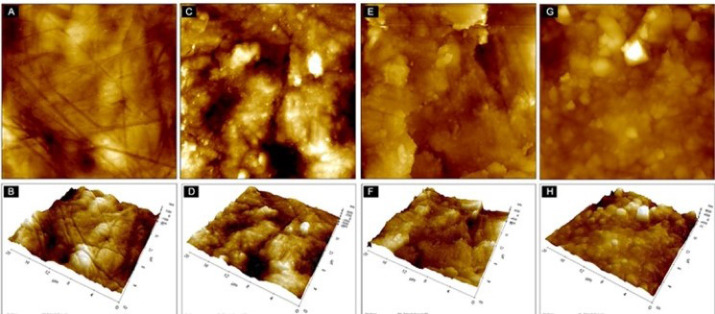
An image from the buccal enamel surface captured by atomic force microscope, showing two-dimensional (upper row) and three-dimensional (lower row) before bracket bonding (A, B), after bracket debonding and resin removal using high-speed 12 flute tungsten carbide bur (C, D), low-speed 12 flute tungsten carbide bur (E, F) and low-speed 30 flute tungsten carbide bur (G, H)

**Figure 3:**
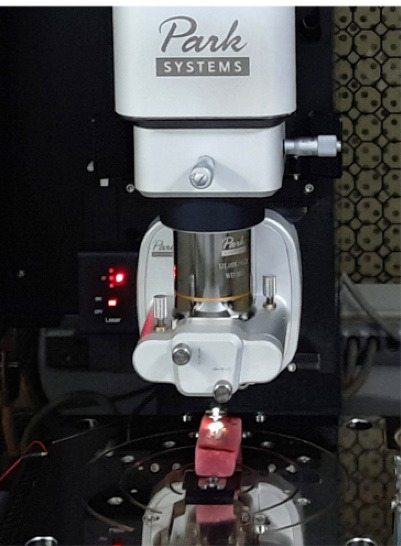
AFM NX20 (Park Systems, South Korea)

All samples (Buccal surface) were evaluated with the AFM before bonding for initial measurements (Figure 2). The AFM NX20 (Park Systems, South Korea) (Figure 3) is attached with a scanner with a maximum range of 100 µm × 100 µm × 5 µm in the x, y and z axes, respectively. The tip of the silicon/silicon nitride probe (TESPA-V2, Bruker AFM probes, Santa Barbara, CA, USA) with a bending constant of k = 42 N/m and a radius tip of 15 nm was brought near the surface of the sample and then moved relative to each other in a raster pattern, and surface roughness is measured at multiple regions across the buccal surface of the enamel specimen. This is called as contact mode of AFM operation. Three various areas were assessed on the enamel surface situated at the centre of the sample. To minimise the error; the mean of these three measurements was taken. The images were taken at 20 µm × 20 µm scan size.

 These measurements include roughness parameters given in nanometres:

- Ra value: Mean roughness values are the arithmetic average deviation of the surface heights (peaks) and depth (valleys) from a centre line of the assessment length
- Rq value: Root mean square roughness is the height deviation parallel to the centre line of assessment length
- Rt value: Maximum roughness height that designates the maximum profile on the surfaces (peak to valley height).^20^

###  Application of adhesive materials

 After mounting, the samples were cleansed with water followed by drying with pressurised air (oil-free). All the samples were etched with 37% ortho-phosphoric acid for 30 seconds, washed and air dried, till a frosty dull white appearance is observed on the etched surface. In groups G and Y, the adhesive primer was coated on the teeth and cured with LED light for 10 seconds, then both were bonded using Transbond XT orthodontic adhesive and in group R, a silane coupling agent along with the same adhesive was used. The brackets were seated in the centre of the tooth with firm pressure. With the help of the sharp explorer, excess flash is removed carefully and cured for 40 seconds as per the manufacturer’s instruction. Then cured specimens were preserved in isotonic saline solution for 7 days at room temperature before debonding. Debonding of the bracket was carried out by debonding pliers with the application of gentle compressing force on the tie wings. The resin leftovers are removed by three different methods, which were as follows:

- Sub-group A: Clean-up with high-speed 12-flute TC bur (10 teeth surfaces per group)
- Sub-group B: Clean-up with low speed 12- flute TC bur (10 teeth surfaces per group)
- Sub-group C: Clean-up with low speed 30 flute TC bur (10 teeth surfaces per group)

After the full removal of the adhesive remnants from the teeth surfaces, the teeth were examined visually under operating light in both wet and dry environments, After that, all the specimens were once again assessed by AFM to determine different parameters of enamel surface roughness i.e. Ra, Rq and Rt. (Figure 2). All the procedures including bonding, debonding, and clean-up were carried out by the primary investigator.

###  Statistical analysis

The surface roughness values of enamel were tabulated and analysed with SPSS version 23 (IBM Statistics, Chicago, USA). The normality assumption of distributed variables was tested by the Shapiro-Wilk test. Roughness parameters (Ra, Rq and Rt) were assessed before bonding and after residual resin, removal using the paired student t-test. Roughness parameters for each group were analysed using ANOVA and for multiple comparisons within the groups, the Tukey test was used. The level of significance of *P* < 0.05 was determined for all analyses.

## Results

Comparing the initial values with values after all the 3 resin removal methods, showed an increase in enamel surface roughness. The roughness parameters (Ra, Rq, and Rt) values in all the 3 groups showed statistically significant changes after enamel clean-up compared with the baseline values (*P* < 0.001) as shown in Table 1.

**Table 1:** Pre- and Post-distribution of Ra, Rq and Rt among study groups

**Parameter**	**Bracket**	**Pre**	**Post**	***P*** ** value**
		**Mean±S.D**	**Mean±S.D**	
Ra	Group G	41.2 ± 7.2	81.5 ± 27.9	< 0.001*
	Group Y	41.1 ± 7.1	112.5 ± 44.2	< 0.001*
	Group R	41.1 ± 7.3	85.5 ± 26.8	< 0.001*
Rq	Group G	61.0 ± 8.0	100.5 ± 36.6	< 0.001*
	Group Y	60.2 ± 9.8	148.6 ± 47.6	< 0.001*
	Group R	61.6 ± 8.0	109.9 ± 35.4	< 0.001*
Rt	Group G	395.9 ± 65.1	803.8 ± 370.7	< 0.001*
	Group Y	395.8 ± 65.3	1325.4 ± 464.4	< 0.001*
	Group R	395.9 ± 65.2	919.8 ± 399.6	< 0.001*

* Significant at 5% level of significance (*P* < 0.01).

Comparison of Ra values in all 3resin removal groups by ANOVA and post hoc Tukey tests were found to be statistically insignificant for all the groups and sub-groups respectively. (Table 2).

**Table 2:** Multiple comparisons of Ra value by 3 different types of resin removal methods

**Bracket**	***P*** ** value**	***P*** ** value**	***P*** ** value**
	**A vs B**	**A vs C**	**B vs C**
Group G	0.441	0.807	0.814
Group Y	0.082	0.935	0.16
Group R	0.516	0.742	0.173

* Significant at 5% level of significance (*P* < 0.01).

Comparison of Rq values in all the 3 resin removal groups by ANOVA exhibited statistically significant differences for group G (*P* = 0.047), and according to Tukey’s post hoc tests, Rq values are highly significant for group G between sub-group A and B (*P* = 0.042) i.e. between high speed 12- flute TC bur and low speed 12- TC bur (Table 3).

**Table 3:** Multiple comparisons of Rq value by 3 different types of resin removal methods

**Bracket**	***P*** ** value**	***P*** ** value**	***P*** ** value**
	**A vs B**	**A vs C**	**B vs C**
Group G	0.042*	0.705	0.203
Group Y	0.272	0.956	0.169
Group R	0.605	0.841	0.915

* Significant at 5% level of significance (*P* < 0.01).

Rt values after resin removal in three groups were compared using ANOVA which showed a highly significant difference for Group G (*P* = 0.006) and statistically significant differences for Group Y (*P* = 0.024). Multiple comparisons of Rt values for Group G showed highly significant differences between Sub-groups A and B (*P* = 0.006) and significant differences between Sub-groups B and C (*P* = 0.049) i.e. between high speed 12-flute TC bur and low-speed 12-flute TC bur and between low-speed 12-flute TC bur and low-speed 30-flute TC bur respectively. Multiple comparisons of Rt values for Group Y showed a significant difference between Sub-groups A and B (*P* = 0.018) i.e. between high-speed 12-flute TC bur and low-speed 12-flute TC bur (Table 4).

**Table 4:** Multiple comparisons of Rt value by 3 different types of resin removal methods

**Bracket**	***P*** ** value**	***P*** ** value**	***P*** ** value**
	**A vs B**	**A vs C**	**B vs C**
Group G	0.006*	0.644	0.049*
Group Y	0.018*	0.23	0.439
Group R	0.769	0.846	0.436

* Significant at 5% level of significance (*P* < 0.01).

## Discussion

Recent advancements in dental materials resulted in superior bond strength between enamel and adhesive, minimizing bracket bond failure rate.^21^ Various debonding methods have been developed to decrease enamel damage. Preserving the outer layer of enamel is of utmost significance for the orthodontist when removing the bracket and the adhesive remnants,^20^ because the outer layer is rich in mineral content and fluoride compared to deep layers. Destruction of the outer layer of enamel may result in reduced enamel resistance and increases the chance of enamel decalcification. Enamel damage was observed with various types of brackets, different retention methods and different resin removal procedures.^22^ Therefore the objective of this research was comparison and assessment of the surface roughness of enamel after debonding of brackets (metal and ceramic) bonded by different techniques and also the effect of three composite removable procedures on enamel surface roughness.

 Debonding and adhesive removal procedures are dependent on operated, so the results might differ among operators. To minimize the error, a single operator performed all the clinical procedures.

In this experimental study, the assessment of surface roughness was subjected to AFM as it is a non-invasive approach and has proven to be an effective analysis for hard surfaces like enamel that illustrates micro-irregularities. Furthermore, AFM gives 3D data at the nanoscale level with perpendicular and asymmetrical resolutions, requiring minimum specimen preparation and also helps to re-examine the sample again.^17,19,22^

Previous studies have used SEM images to assess surface roughness; although, this method can provide only qualitative data i.e.; subjective information.^23^ This cannot be utilized for comparative and correlative assessment of surface roughness between the groups. Thus, this study has an advantage as quantitative measurements from AFM help in better comparative assessment between groups.

From the results, it was found that irrespective of the adhesive removal methods used (bur type), an increase in surface roughness values of enamel was observed after resin removal therefore none of the adhesive removal methods can fully restore the enamel to its earlier morphology as found in previous studies.^13,24^ Sigilião et al^25^ stated that all rotary instruments lead to alternation in enamel morphology.

TC burs are the most commonly used adhesive removal burs by clinicians.^20,26^ TC burs have different shapes and various blade types. The regularly operated TC burs have 8-30 flutes. 12-30 flute TC burs were considered to cause less damage to enamel.^24^ Thus 12 and 30 flute TC burs were evaluated in this experimental study.

In this study after the residual resin removal between 12 flute TC bur at high-speed and 12 flute TC bur at low-speed it was observed, an increase in surface irregularities on the enamel surface which were significant for Rq (group G, Table 3) and Rt (group G and group Y, Table 4) but not for Ra values. This outcome showed an irregular surface with high vertical peaks and sporadic deep-rooted scratches, representing the severe mechanical enamel destruction which could not be detected by Ra, as Ra illustrates only average roughness and fails to describe the peak height and depth.^25^ The findings were in agreement with those of Ahrari et al^11^ and Hannah and Smith.^23^ They have highlighted that low-speed TC burs would be less deleterious to the adjacent enamel compared to high-speed TC burs for resin removal.

Previous studies recommend the usage of low-speed 12-flute TC burs, which generate minute scratches with a lesser level of enamel loss^1,12,27-29^ which follows our study.

Multiple comparisons of Rq and Rt values in metal brackets with mechanical retention showed more enamel surface irregularities with high-speed 12 flute TC but when compared with low-speed 12 flute TC bur. Similar findings were observed in the ceramic bracket with mechanical retention for Rt. Rt values in the ceramic bracket with chemical retention showed more enamel surface roughness with low-speed 30 flute TC bur when compared with low-speed 12 flute TC bur. This study is not in agreement with Campbell et al^30^ who suggested that 30 flutes TC bur was the most efficient procedure of highly filled resin removal with less quantity of enamel scar, this concordance in the study might be due to the variation in resin removal methods and the type of investigation carried out as earlier was an SEM study.

Radlanski^28^ reported that there was an increase in surface roughness parameters at Ra and Rz along Y-axis with 5-blade TC bur compared to the 30-blade group, it might be due to the cutting blades which are in a perpendicular direction at the scanned area, this was in accordance with our study.

In our study, the variation in burs cutting efficiency might be due to various factors which include, bur rotation speed, number of blades, pressure against the enamel during resin removal, and amount of time spent by the operator for the resin removal, that influence the changes that are observed here.^20,21^

Bicakci et al^31^ previously advocated the use of burs at high speed in absence of water coolant. Subsequently, it resulted in heat in the pulp chamber followed by hyperaemia and sometimes ruptures of odontoblasts. Although, this phenomenon is temporary and reversible, thereby indicating healing of pulp tissue might occur within 20 days. Thus in this study, water cooling was used along with the TC burs during the resin removal procedures.

This experimental study was conducted with laboratory conditions, so it is impossible to mimic the oral condition. The bond strength in in-vitro studies does not completely represent the oral environment. Although, with an increase in the number of samples, and the data showing parametric distribution, this standard experimental method was created to produce a laboratory condition that was as similar to the clinical scenario as possible, and thus extrapolation of the above in-vitro findings to the clinical situation is recommended for further in vivo and ex vivo investigations.^32^

## Conclusion

 Based on the results it could be concluded that:

1. None of the composite removal methods was capable of re-established enamel to its earlier morphology.
2. Application of low-speed 12 flutes TC bur found to be proven and the safe procedure for adhesive remnants removal irrespective of metal or ceramic brackets bonding techniques.
3. An incremental increase in surface roughness parameters was reported after composite removal with a high-speed 12 flute TC bur, particularly for Rq and Rt measurements and which was statistically significant. However, the enamel loss observed was clinically insignificant, composite remnants removal by a high-speed 12 flute TC bur should be performed carefully.
4. Application of low-speed 30 flute TC bur showed a moderate amount of surface roughness parameters in comparison with the other two burs.

## Competing Interests

 No conflicts of interest.

## Ethical Approval

 The study was approved by the institutional ethics committee in Albadar Rural dental college and hospital, Karnataka, India.

## Funding

 No funding sources.
